# Recommendations for the prevention of fragility fractures: a consensus from international experts and Ibero-American scientific societies

**DOI:** 10.1007/s11657-025-01551-2

**Published:** 2025-06-12

**Authors:** Pilar Sáez-López, César Aldecoa Álvarez-Santullano, Rosa Arboiro-Pinel, José Luis Baquero Úbeda, José Carlos Bastida Calvo, Francisco Baixaulí García, Concepción Cassinello Ogea, Patricia Ysabel Condorhuamán Alvarado, María Cortés Berdonces, Leonor Cuadra Llopart, Nuria Fernández Martínez, Mercé Giner García, Rafael Manuel Micó Pérez, Blanca Mur Molina, Antonio Naranjo Hernández, José Luis Neyro Bilbao, Cristina Ojeda-Thies, Santiago Palacios Gil Antuñano, Manuel Santiñá Vila, José Soto Bonel, Francisco José Tarazona-Santabalbina

**Affiliations:** 1 Hospital La Paz Research Institute (IdiPAZ), Madrid, Spain; 2 Department of Geriatrics, Hospital Universitario Fundación Alcorcón, Madrid, Spain; 3 Department of Anesthetics and Reanimation, Rio Hortega University Hospital, Valladolid, Spain; 4 Department of Internal Medicine, Hospital Universitario Fundación Jiménez Díaz, Madrid, Spain; 5 International Relations and Access, OAFI, Madrid, Spain; 6 National Group on Osteoarticular Disease and Osteoporosis, Sociedad Española De Médicos Generales y de Familia (SEMG), Madrid, Spain; 7 SECOT – Sociedad Española de Cirugía Ortopédica y Traumatología, Hospital Universitario y Politécnico La Fe, Valencia, Spain; 8 Department of Anesthesiology, Reanimation and Pain Treatment, Hospital Universitario Infanta Sofía, Madrid, Spain; 9 Universidad Europea de Madrid, Madrid, Spain; 10 Department of Geriatrics, Hospital Universitario La Paz, Hospital La Paz Research Institute (IdiPAZ), Madrid, Spain; 11 Universidad Autónoma de Madrid, Madrid, Spain; 12 Department of Endocrinology, Hospital Universitario Ruber Juan Bravo, Madrid, Spain; 13 Department of Medicine, Faculty of Biomedical and Health Sciences, Universidad Europea de Madrid, Madrid, Spain; 14 Department of Geriatrics, Hospital Universitario de Terrassa, Consorci Sanitari de Terrassa, Barcelona, Spain; 15 Actium Functional Anatomy Research Group, Universitat Internacional de Catalunya, Barcelona, Spain; 16 Department of Geriatrics, Hospital Universitario de Toledo, Toledo, Spain; 17 Sociedad Española de Investigación Ósea y del Metabolismo Mineral (SEIOMM), Bone Metabolism Unit, Internal Medicine Clinical Management Unit, Virgen Macarena University Hospital, Seville, Spain; 18 Department of Cytology, and Normal and Pathologic Histology, University of Sevilla, Seville, Spain; 19 SEMERGEN Foundation, Madrid, Spain; 20 Xativa-Ontinyent Health Department, Health Center Fontanars dels Alforins, Valencia, Spain; 21 Department of Physical Medicine and Rehabilitation, Hospital Universitario Ramón y Cajal, Madrid, Spain; 22 Department of Rheumatology, Hospital Universitario de Gran Canaria Dr. Negrin, Las Palmas de Gran Canaria, Spain; 23 Universidad de Las Palmas de Gran Canaria, Las Palmas de Gran Canaria, Spain; 24 Academy of Medical Sciencesof , Bilbao, Spain; 25 International Master of Climaterium and Menopause, Universidad de Madrid (UDIMA), Madrid, Spain; 26 Sociedad Iberoamericana de Osteología y Metabolismo Mineral (SIBOMM), Lisbon, Portugal; 27 Department of Traumatology and Orthopaedic Surgery, 12 de Octubre University Hospital, Avenida de Córdoba S/N, 28041 Madrid, Spain; 28 Department of Surgery, School of Medicine, Complutense University of Madrid, Madrid, Spain; 29 Clínica Palacios Salud de La Mujer, Madrid, Spain; 30 Institut de Investigacions Biomèdiques August Pi I Sunyer (IDIBAPS), Barcelona, Spain; 31 Fundación Instituto San José, Madrid, Spain; 32 SEDISA - Sociedad Española de Directivos de La Salud, Madrid, Spain; 33 Geriatric Medicine Department, Hospital Universitario de La Ribera, Alzira, Spain; 34 Medical School, Universidad Católica de Valencia San Vicente Mártir, Valencia, Spain; 35 Centro de Investigación Biomédica en Red Fragilidad y Envejecimiento Saludable (CIBERFES), Madrid, Spain

**Keywords:** Osteoporosis, Fragility fracture, Consensus statement, Stakeholder

## Abstract

**Purpose:**

To develop a multidisciplinary consensus outlining key recommendations to prevent fragility fractures and improve care through coordinated efforts across healthcare sectors.

**Methods:**

An international group of experts, coordinated by the Spanish National Hip Fracture Registry (RNFC), engaged over 300 professionals and 31 scientific societies. Using a nominal group technique, the committee reviewed scientific evidence and collaboratively developed ten core recommendations. The consensus was refined through multiple telematic reviews and finalized at the 7th RNFC Annual Meeting in March 2024.

**Results:**

The consensus presents ten actionable recommendations: (1) inclusion of osteoporosis and fragility fractures in health policies, (2) early detection and management of frailty and falls, (3) implementation of clinical practice guidelines, (4) promotion of fracture registries and audits, (5) support for Orthogeriatric Units and Fracture Liaison Services (FLS), (6) adoption of a “Fragility Fracture Code,” (7) empowerment of Primary Care in fracture prevention, (8) increased patient association involvement, (9) public awareness campaigns, and (10) promotion of research including patient-reported outcomes.

**Conclusions:**

Fragility fractures are a major public health issue with rising incidence, disability, and healthcare costs. This consensus offers unified, evidence-based guidance for policy makers, healthcare professionals, and patient organizations. Broad dissemination and implementation of these recommendations aim to reduce fracture rates and enhance patient outcomes through coordinated, multidisciplinary care.

**Supplementary Information:**

The online version contains supplementary material available at 10.1007/s11657-025-01551-2.

## Introduction

Fragility fractures have very relevant consequences for the health system and important preventable causes, of which perhaps osteoporosis is the most important, though other entities such as osteomalacia and renal osteodystrophy also lead to bone fragility. Osteoporosis is a systemic skeletal disease characterized by a low bone mass and deterioration of bone microarchitecture, leading to bone fragility and an increased risk of fractures [1]. Further contributing factors exist to fractures occurring in the setting of osteoporosis, such as frailty, age, and falls. Both osteoporosis as well as its direct consequence, fragility fractures, increase exponentially with age, most markedly over the age of 75 [2]. According to the SCOPE 21 report, the prevalence of osteoporosis was around 5.4% in Spain in 2019, similar to the European Union average (5.6%). A 30% increase is expected in the annual number of fractures by 2034, with an estimated 370,000 cases per year [2].

Fragility fractures lead to significant functional impairment, with only 40% of patients suffering fractures recovering their previous quality of life, autonomy and independence [3]. They are a cause of increased mortality as well, estimated at 74 deaths after suffering a fracture per 100,000 persons aged 50 and older, a figure that is even higher for hip fractures [2, 4]. Having suffered a previous osteoporotic fracture increases the risk of new fractures 1.5–2 times [5], and is one of the most important predictors of subsequent fractures, together with age [6]. The total healthcare cost associated with fragility fractures in Spain was 4300 million euros (€) in 2019, or 3.8% of healthcare expenditure, above the European average of 3.5% [2]. Scientific evidence on the efficacy of treatment for fracture prevention is readily available. Real life studies have shown that women who follow their osteoporosis treatment reduce their risk of new fractures by 25%, compared to women not treated [7].

There is a great disparity in the approach to osteoporosis and fracture between medical specialties, both in primary and secondary prevention. Each scientific society publishes its guides and recommendations separately, while public health policies lack a coherent common thread and well-defined objectives.

Following the initiative of the RNFC (Spanish National Hip Fracture Audit, or *Registro Nacional de Fracturas de Cadera*), an international group of experts from different disciplines and representatives of 31 scientific societies, working groups and patient associations (Table 1) have agreed on a consensus titled “Recommendations for the Prevention of Fragility Fractures”.

**Table 1:** Scientific societies and social entities participating in the development of this consensus statement

Association (Acronym)/Translation	Country/Region
*Asociación Argentina para el Estudio del Climaterio* (AAPEC)/Argentinian Association on the Study of Menopause	Argentina
*Academia de Ciencias Médicas de Bilbao* (ACMB)/Academy of Medical Sciences of Bilbao, Spain	Spain
*Asociación Española con la Osteoporosis y la Artrosis* (AECOSAR)/Spanish Association (of patients) with Osteoporosis and Osteoarthritis	Spain
*Asociación Latinoamericana de Endocrinología* (ALEG)/Latin American Association of Endocrinology	Latin America
*Academia Mexicana de Geriatría A.C* (AMG)/Mexican Academy of Geriatrics	Mexico
*Asociación Colombiana de Menopausia* (ASOMENOPAUSIA)/Colombian Menopause Association	Colombia
*Fundación Navarro Viola* (FNV)/*Navarro Viola Foindation*	Argentina
*Fundación Trauma* (FT)/Trauma Foundation	Argentina
Fragility Fracture Network Denmark (FFN-Denmark)	Denmark
Fragility Fracture Network Greece (FFN-Greece)	Greece
Fragility Fracture Network Portugal (FFN-Portugal)	Portugal
*Fundación Hispana de Osteoporosis y Enfermedades del Metabolismo Óseo* (FHOEMO)/Hispanic Foundation of Osteoporosis and Bone Mineral Metabolism Disease	Spain
Osteoarthritis Foundation International (OAFI)	Spain
*Red Argentina de Fracturas de Cadera* (RAFCA)/Argentinian Network of Hip Fractures	Argentina
*Registro Nacional de Fracturas de Cadera* (RNFC)/Spanish National Hip Fracture Registry	Spain
*Sociedad Española de Calidad Asistencial* (SECA)/Spanish Society Of Quality of Care	Spain
*Sociedad Española de Cirugía Ortopédica y Traumatología* (SECOT)/Spanish Society of Orthopedic Surgery and Traumatology	Spain
*Sociedad Española de Anestesiología, Reanimación y Terapéutica del Dolor* (SEDAR)/Spanish Society fo Anesthesiology, Reanimation and Pain Therapy	Spain
*Sociedad Española de Directivos de la Salud* (SEDISA)/Spanish Society of Health Directors	Spain
*Sociedad Española de Endocrinología y Nutrición* (SEEN)/Spanish Society of Endocrinology and Nutrition	Spain
*Sociedad Española de Fracturas Osteoporóticas* (SEFRAOS)/Spanish Society of Fragility Fractures	Spain
*Sociedad Española de Geriatría y Gerontología* (SEGG)/Spanish Society of Geriatrics and Gerontology	Spain
*Sociedad Española de Investigación Ósea y del Metabolismo Mineral* (SEIOMM)/Spanish Society of Bone and Mineral Metabolism Research	Spain
*Sociedad Española de Medicina Geriátrica* (SEMEG)/Spanish Society of Geriatric Medicine	Spain
*Sociedad Española de Médicos de Atención Primaria* (SEMERGEN)/Spanish Society of Primary Care	Spain
*Sociedad Española de Médicos Generales y de Familia* (SEMG)/Spanish Society of General and Family Physicians	Spain
*Sociedad Española de Medicina Interna* (SEMI)/Spanish Society of Internal Medicine	Spain
*Sociedad Española de Reumatología* (SER)/Spanish Society of Rheumatology	Spain
*Sociedad Española de Rehabilitación y Medicina Física* (SERMEF)/Spanish Society of Rehabilitation and Physical Medicine	Spain
*Sociedad Iberoamericana de Osteología y Metabolismo Mineral* (SIBOMM)/Iberian-american Society of Osteology and Mineral Metabolism	Ibero- America
Asociación Vasca de Geriatría y Gerontología (ZAHARTZAROA)/Basque Association of Geriatrics and Gerontology	Spain

This paper aims to disseminate this multidisciplinary consensus, justifying each of the recommendations for fracture prevention, referencing current scientific evidence, so that all the agents involved (healthcare administration, professionals, scientific societies, patient associations and society in general) can apply the recommendations to achieve a reduction in the incidence of osteoporosis and of fragility fractures.

## Methods

The initiative to create a consensus to enable fragility fracture prevention stems from the RNFC, a multidisciplinary working group of 300 professionals from 105 hospitals and several disciplines who voluntarily work to improve the care of hip fracture patients through audit. The method followed was a nominal group process: first, members of the RNFC scientific committee performed a literature search on general measures to improve the prevention of fragility fractures (defined as one that results from low-energy trauma, such as a fall from standing height or less), synthesized the results and prepared an initial provisional document containing the proposals.

This document was shared with scientific societies and fracture-related patient associations who had previously endorsed the RNFC and are included as stakeholders in RNFC publications and newsletters, and a nominal expert committee was set up. The expert delegates, as well as the scientific societies they represent, are specialized in different aspects of hip fracture care, so they were asked to review the complete document, but particularly the recommendations most related with their knowledge area, so their opinion bore more weight in case of controversy (for example, the recommendations regarding the role of the primary care physician was reviewed by the scientific societies representing primary care, and the content describing patient education and participation was revised by patient associations; scientific societies representing geriatrics revised aspects related to frailty, falls and orthogeriatrics, while those studying bone metabolism and rheumatology reviewed statements discussion fracture liaison services (FLS), etc.).

Throughout the end of 2023 and first trimester of 2024 and via telematic means, the experts conducted a review process to agree on key preventative measures and drafted a consensus including the ten most important measures in the medical, political, and social spheres. The decision to limit the recommendations to ten was through agreement among the participating experts, to synthesise the most important and feasible proposals into a decalogue.

This document was presented during the 7 th Annual Meeting of the RNFC, on March 8 th, 2024, to which the expert delegates and members of 20 Spanish scientific societies as well as RNFC participants were invited to discuss each point of the decalogue. After the contributions from the meeting and taking the review and endorsement of international scientific societies into account, a consensus outline with the recommendations for fracture prevention was drafted and shared on the webs of several participating societies and associations. Once the recommendations had been agreed upon and published, the experts continued with their telematic work for a further eight months to finish the writing of the complete manuscript justifying the expert recommendations, again distributing the elaboration of each recommendation to the most expert delegates in each particular aspect.

### Justification of the expert recommendations for the prevention of fragility fractures

**Recommendation 1** **:** **Fragility fractures should be included in international, national, and regional health** **policies, plans and strategies, with the aim of preventing new fractures and their serious consequences. Early diagnosis and prevention strategies of osteoporosis and fragility fractures should be promoted, based on the presence of risk factors.**

In Spain, 3 million people have been diagnosed with osteoporosis, but many more have osteopenia, a previous stage in the loss of bone mass [8]. In many cases, osteoporosis is discovered late, after an unexpected fragility fracture. Approximately 65% of people suffering fragility fractures have not been diagnosed with osteoporosis [9]. A new fragility fracture occurs every two minutes in Spain [10]. From the moment of the fracture, a certain level of chronic pain and disability will follow the patient, to a greater or lesser extent. Thus, it can be concluded that it is a serious disease, very prevalent — particularly in postmenopausal women — that not only places the patient’s health at risk, but also affects quality of life and that is frequently diagnosed and approached late [2, 3, 8, 9].

It is therefore of capital interest to incorporate osteoporosis as a chronic disease in health policies, plans and strategies, on a regional, national and international level.

**Recommendation 2: Frailty should be detected early, and appropriate interventions carried out to try to revert it** **and raise awareness towards falls prevention, particularly in older individuals.** **The prescription of individualized therapeutic exercise should be promoted** **and** **Falls Prevention Units implemented,** **with the aim of a multidisciplinary approach tailored for each patient.**

The “Consensus Document on the Prevention of Frailty and Falls in Older Persons” approved in 2014 by the Interterritorial Council of The Spanish National Healthcare System and updated in 2022, focuses on addressing frailty and the need for early detection and intervention through the healthcare system [11].

Falls are highly prevalent in older persons, multicausal and have devastating consequences, therefore it is important to detect the risk with quick and simple validated tools, including gait and balance tests. Clinical Practice Guidelines for falls prevention recommend individualized, consensual and multifaceted interventions. Review of medications, multicomponent exercise, review of visual acuity and footwear, and modification of architectural barriers in the environment are recommended to prevent falls. The indication for treating osteoporosis and the cardiovascular status should be evaluated, and Falls Prevention Units are an ideal care model to achieve a reduction in falls risk, so their implementation would be advisable [12, 13].

Osteoporotic fractures are more common in frail older patients at risk for falls. Up to two thirds of patients suffering hip fractures are estimated to be frail, and a quarter of them are in their last year of life. Implementation of the recommendations of the Spanish Ministry of Health on the prevention of frailty can contribute to preventing falls and fractures [11]. Performing a comprehensive geriatric and frailty assessment of hospitalized patients with surgical fractures and using scales such as the Clinical Frailty Scale (CFS) — able to predict an increased length of stay, number of complications and 30-day survival — can aid the healthcare team in clinical decision-making. Thus, a greater knowledge is obtained on the potential risks during hospitalization, both in terms of outcomes and survival [14, 15].

Individualized multicomponent exercise is effective to prevent falls and reduce fracture risk. Age is not a good marker of frailty as an individual’s usual physical activity. Limiting sedentary time already increases bone mineral density [12]. Targeted therapeutic exercise can improve mobility and gait speed in patients with hip fracture, during acute hospitalization as well as after discharge. Specifically, re-education of gait, balance and functional tasks are especially effective. In this regard, some studies recommend resistance exercise while others question the efficacy of aerobic training [16, 17].

**Recommendation 3** **:** **Clinical practice guidelines for patients with high risk of fracture should be implemented,** **enabling adequate and uniform diagnosis, treatment and follow-up of patients who have suffered an osteoporotic fracture.**

Throughout a single care process, there are several points where diagnostic or therapeutic controversies may exist. Thus, it is common for different professionals to adopt very diverse approaches for the same problem [18].

Clinical practice guidelines (CPG) are documents that describe the best practices and recommendations to manage a disease or specific medical condition, systematically and based on the best available evidence. Thus, they provide systematic recommendations to aid healthcare professionals in clinical decision making, improving quality of care and reducing unwarranted variability in clinical practice [19].

Implementation of CPG is essential to ensure consistent and adequate diagnosis, treatment and follow-up of patients at risk for osteoporotic fracture or who have already suffered a fragility fracture. Following CPG homogenizes clinical care, helps maintain geographic and social equitableness, improves health outcomes through prompt identification of fracture risk, and provides a greater quality of care for patients by improving treatment adherence and optimization of healthcare resources. All while also identifying areas of improvement in the setting of research and development of preventative health plans [20]. In addition, CPG reduce unjustified variation of patient care depending on whether they are treated in primary care or in the hospital.

**Recommendation 4: The creation and/or participation in comprehensive fracture registries and audits** at the national as well as regional level should be promoted.

Clinical practice guidelines are useful for suggesting what should be done to clinically manage each patient process, but registering each therapeutic decision and the patient’s evolution tells us what really happened and helps evaluate quality of care. Adequate management of fractures, especially hip fractures, requires integrating excellent care from nurses, anaesthetists, orthopaedic surgeons and other specialties such as geriatrics, internal medicine and physical medicine and rehabilitation. Ample evidence exists on the usefulness of quality standards to improve care, as has been proven for orthogeriatric collaboration and fracture liaison services [21].

Spain has registries with regional and national coverage, such as those offered by SEIOMM (*Sociedad Española de Investigación en Metabiolismo Mineral y Óseo, Spanish Society for Research in Bone and Mineral Metabolism*) with its REFRA (*Registro Español de Fracturas, Spanish Fracture Registry*) or by the RNFC itself with the Spanish National Hip Fracture Registry.

REFRA is an online information service platform owned by SEIOMM with the main goal of creating a multicentric registry of the epidemiologic, clinical, functional characteristics of patients with fragility fractures, as well as the care provided and their follow-up. The information obtained by this registry will provide epidemiologic data on fragility fractures in Spain and evaluate outcomes over time, allowing for comparisons between geographic areas, autonomous regions and internationally [22].

The Spanish National Hip Fracture Registry (*Registro Nacional de Fracturas de Cadera, RNFC*) is more specific and enables hospitals to compare the care provided with clinical standards. It is an audit with access to information and continuous feedback that makes it possible for organizations to detect deficits and implement improvements and, simultaneously, to evaluate the impact of these measures. The benefits of this type of registry explains the existence of similar hip fracture registries in Australia and New Zealand, Denmark, Ireland, Italy, Mexico, the Netherlands, Norway, Scotland, South Korea, Spain, Sri Lanka, Sweden and the unified database of England, Northern Ireland and Wales [23].

The RNFC collects information on the characteristics of patient aged 75 years and older who have suffered hip fractures and the care provided during acute hospitalization and the clinical course for one month after the fracture [24]. Three hundred professionals from over 100 Spanish hospitals participate in the registry, which has received the endorsement of 29 scientific societies, as well as recognition by the Spanish Ministry of Health as “Health Registry of Interest for the National Healthcare System” [25].

Over the past six years, the hospitals participating in the RNFC have improved quality indicators though a more prompt surgery, better postoperative mobilization and functional recovery, reducing pressure sores and increasing prescription of bone-protective medication, all elements that help improve these patients’ clinical course and recovery [26].

**Recommendation 5:** **The creation of Orthogeriatric Units and/or Multidisciplinary Shared Care Teams (with clinicians of any specialty with expertise in this process) to improve the care provided and health outcomes of older patients hospitalized with fragility fractures should be supported. Fracture Liaison Services (FLS) should be improved and their numbers reinforced to guarantee integrated and multidisciplinary care of these patients, ensuring detection, evaluation and appropriate treatment of patients who have suffered a first osteoporotic fracture. This type of units includes structured, multidisciplinary, agile and continuous care – involving primary care, specialized care, nursing care, community pharmacies and rehabilitative care, among others –, apply clinical practice guidelines and use registries as well as validated quality indicators.**

The preoperative care of a patient with a hip fracture by a multidisciplinary team including clinicians experienced in treating older, frail patients, the complications of trauma and surgery and bone health (teams known as Orthogeriatric Units or Shared Care Teams) offers the opportunity to diagnose osteoporosis, evaluate frailty and falls risk, and to prescribe a personalized treatment effective at preventing new fractures [27]. Several studies and meta-analyses have shown that Orthogeriatric Units based on multidisciplinary teams expert in managing patients with hip fractures increase the percentage of patients operated promptly, reduce complication rates and mortality, improve functional recovery, prevent falls and increase prescription of osteoprotective medication [28]. They reduce hospital length of stay and readmissions as well and are estimated to be cost-effective for the healthcare system, and are considered a structural quality indicator by several registries and clinical practice guidelines [29].

Fracture Liaison Services, the majority of which are outpatient, are effective regarding the identification, evaluation, and treatment of patients with fragility fractures, compared with usual care. Some systematic reviews conclude that they improve integrated care, including the fracture as well as comorbidities, leading to a reduction in mortality and postoperative complications. They also reduce length of stay and readmissions, which would suggest a faster recovery, improving the patients’ functionality and quality of life and liberating hospital resources. Furthermore, they reduce the absolute risk of refracture and increase treatment adherence [30–32].

On the other hand, the use of quality tools such as the United Kingdom and New Zealand Clinical Standards established for FLS Units and endorsed by recognised entities such as IOF, FFN and the Bone Health and Osteoporosis Foundation, whose main goal is the prompt detection and treatment of patients aged 50 and older suffering fragility fractures [33–35]. In addition, the Spanish Society for Healthcare Quality (*Sociedad Española de Calidad Asistencial, SECA*) has developed an accreditation standard for FLS Units. The standard aims to help the implementation of the care model by establishing a defined set of criteria and sub-criteria [36].

Regarding efficiency of FLS, Markov simulation models have proven they are cost-effective and that the investment (including costs for personnel, diagnostic procedures, and treatments) is offset by the high cost of fracture care and the reduced quality of life due to fractures, demonstrating a high return of investment [37–40].

**Recommendation 6** **: A** **“Fragility Fracture Code”** **should be created, that would facilitate implementation of multidisciplinary, multimodal processes (or clinical pathways) based on evidence and clinical practice guidelines for each type of fracture. Actions should encompass** **from the scene of injury to the patients’ social and familial reinsertion in their usual environment** **with the maximum independence possible, and prevention and/or treatment of frailty and new fractures. Monitoring and transparent audit of outcomes at the national level should be strengthened.**

Coding patients with diseases in which a delay of care can be key to achieve good results or disabilities is one of the major organizational advances of the health system in the last two decades. Some examples of these codes would be myocardial infarction, acute cerebrovascular disease and sepsis at the level of hospital care, and prevention of cardiovascular disease at the primary care level [41].

Fragility fractures are common, lead to significant morbimortality and dependence, and it is important to provide prompt care, which would justify introducing this code, especially in the case of hip fractures [2, 3].

Implementing a “Fragility Fracture Code” enables quick identification and intervention of the acute fracture as well of secondary prevention of new fragility fractures, including periprosthetic/periimplant fractures [42, 43]. This code should be established the moment the patient is diagnosed, independently of the healthcare setting in which the diagnosis is made, and should continue along the established pathway to ensure recovery and prevent new fractures.

The “Hip Fracture Code” defines the hip fracture care process, including transport to the hospital, pain management and patient optimization, facilitating prompt surgery, reducing pain, complications, dependence, institutionalization, costs and mortality [44]. The time standards include arrival at the emergency department less than two hours after the fall, provision of analgesic medication in less than 30 min after arrival, spending less than four hours in the emergency department, receiving surgery in less than 48 h and being mobilized in less than 72 h after initial contact [45, 46].

To ensure standards are met, the following systemic conditions must be fulfilled:

To ensure standards are met, the following systemic conditions must be fulfilled:

- Training of personnel to facilitate **early recognition** of hip fractures
- Consideration of the fracture as an **urgent medical and surgical entity**
- Prioritization of transfers in an adequate transport resource
- Coordination between the pre-hospital emergency services, hospital emergency department, orthopaedic surgery, medical services, and anaesthesia.
- Availability of resources, orthogeriatric and specific care every day of the week, enabling surgical management in 24 to 48 h and initiation of rehabilitative care 72 h after initial contact.

**Recommendation 7** **: The** **Primary Care Professionals’ role in diagnosis and follow-** **up should be boosted in all phases of the care process of patients with low bone mass, particularly regarding identification and management of people with a high risk of falls and subsequent osteoporotic fractures, in order to guarantee therapeutic adherence and persistence for the time required.**

There is a high prevalence of fragility fractures among patients aged 70 and older treated in Primary Care in Spain (17.7%), and among these, a diagnostic and treatment gap exists of 34.3% and 41.2%, respectively [47]. It is imperative for Primary Care Physicians to be aware of risk factors for fragility fractures, to undertake a physical exam reviewing elements such as changes in stature or spinal curvature, to order laboratory tests with parameters of bone and mineral metabolism, conventional radiographs and densitometries. The Primary Care Physician should also be familiar with risk stratification tools like FRAX (Fracture Risk Assessment Tool), that help to individualize the patients’ management.

Primary Care Professionals provide education on healthy lifestyles that are vital for prevention as well as treatment of osteoporosis, including endorsing a diet rich in calcium and vitamin D, promoting physical activity, eliminating toxic habits (alcohol, tobacco), evaluating falls risk (screening for frailty, review of medications, removal of obstacles at home, etc.), and controlling height and weight. Communication with patients should also be maintained, to involve them in the importance of their disease and establish the necessary and timely measures to reduce their risk. Preventative treatment should be individualized, based on individual fracture risk, age, prior history, consideration of risk factors and densitometric findings, and the therapeutic goal is the reduction of fractures [48].

Likewise, the Primary Care Physician should plan the patients’ follow-up to evaluate treatment efficacy, safety and adherence [49]. Coordination with FLS, if they exist locally, or with other levels of care involved (Departments of Endocrinology, Rheumatology, Internal Medicine, Geriatric Medicine) is desirable to ensure a comprehensive and multidisciplinary approach to the treatment of osteoporosis. The comprehensive vision and management of osteoporosis provided by Primary Care leads to patients receiving effective and preventative treatment over time [50, 51].

**Recommendation 8:** **Participation of patient organizations should increase, taking advantage of their potential (expert patients, complement of service portfolios, etc.) and enabling advice and education of patients regarding their disease, healthy habits, nutrition, physical activities, and treatment adherence. Patients should participate in decision-making regarding their treatment, together with healthcare professionals, thus increasing their quality of life, prioritizing active and healthy ageing.**

The main objective of healthcare management is providing value to the patients, keeping in mind that value is created through interaction between patients and professionals; results should be measured, including outcomes and experiences perceived by the patients themselves (PROMs, patient reported outcome measures, and PREM, patient reported experience measures), as well as costs [52, 53].

In any case, the uncertainty of disease itself entails difficult decisions that include risks, requiring collaboration on all levels (administration, professional and patient) to improve quality of care [54].

Patient participation is backed by several laws such as Spain’s Law 14/1986 of April 25 (Ministry of Presidency, Justice, and Relations with the Courts, 1986), which requires in its articles 4, 5 and 53 that public health services be organized in a participative manner; and article 10.10 recognizes the citizens’ right to participate in healthcare activities. Furthermore, Spain’s Law 41/2002 of November 14 (Ministry of Presidency, Justice, and Relations with the Courts, 2002), which regulates patient autonomy, demands adequate information and patient consent, as well as respect of decisions made freely and voluntarily by patients [55, 56].

**Recommendation 9:** **National, regional and regional campaigns should be promoted, that inform and raise awareness regarding primary and secondary prevention of osteoporotic fractures.**

In the context of patient autonomy, it is imperative that patients are well informed for their self-care and control of their health. A recent study promoted by the Osteoarthritis Foundation International (OAFI) found that the information provided to patients by personnel of the national health system was an area of improvement, and proposed webpages of scientific societies and patient organizations as complementary sources of information [57].

Promoting sensibilization campaigns to inform and raise awareness of osteoporosis requires an integrated and multifaceted approach. First, it is essential to use several forms of media such as television, radio, written press, and internet to disseminate clear and precise information about the disease. Social media is a powerful tool to reach a wide and diverse audience; regular posts, educational videos and patient testimonies can make the topic more visible and easier to understand. Spreading these actions throughout the Latin America, which has a common language and media, can add to this dissemination, thus unifying criteria on managing this disease.

Organization of community events such as health workshops also allows direct interaction with citizens, providing opportunities for answering questions and distributing educational material. Schools and workplaces can be strategic points for giving educational talks, promoting healthy habits from an early age as well as at work. Including early detection programs and free checkups in these campaigns can help identify cases at risk in a timely manner and adopt preventative measures.

Lastly, it is crucial to involve governmental and non-governmental organizations to provide support financing and broadcasting these campaigns, ensuring a greater reach and sustainability over time. The combination of all these efforts can significantly contribute to preventing and treating osteoporosis.

**Recommendation 10:** **Scientific evidence should be generated through quality research on the best way to prevent and treat fragility fractures, including the patients’ perspective, as well.**

Developing clinical research on the prevention and management of fragility fractures is crucial, as they pose a significant public health concern. Despite the solid implementation of the two main models of fracture care in Spain, orthogeriatric and bone metabolism, further research is needed regarding management and results in different organizational environments which would help develop and validate more effective prevention strategies, thus reducing the incidence of these fragility fractures. Exploiting and disseminating the two most prominent fracture registries — RNFC and REFRA — would allow us to achieve these goals [22, 26].

Analysis of the most efficient models of care after a fracture, in public as well as in private healthcare systems, would be of great help for managers in their decision-making, and would provide more evidence to guide clinical practice and health policies.

Risk factors and groups of the population that could benefit of personalized interventions can be identified through research, thus improving the efficiency of health resources and the effectiveness of said interventions. The code “Fragility fracture” in electronic health records could demonstrate the osteoporosis treatment gap to prevent new fractures. Its implementation would act as an indicator of different fracture prevention programs and contribute to real world evidence. National-level data is needed regarding patient satisfaction, quality of life, knowledge about the disease and repercussions of the different fracture prevention models on treatment adherence compared to usual care.

The reputation of several Spanish FLS and orthogeriatric and shared care units should be taken advantage of, with the aim of improving quality and efficiency of all related activities, at the hospital level as well as in primary care. In that sense, active endorsement of research by the administration and scientific societies is both convenient and necessary, to prevent the first fracture from occurring as well as in secondary prevention. Consideration of patient-reported outcomes and experiences (PROMs and PREMs) is key to evaluate the quality and performance of all healthcare services and systems, but their inclusion and implementation is lowdue to several barriers [48].

## Conclusions

- Fragility fractures are a prevalent entity with a growing incidence, that lead to an increased morbidity and mortality among patients who suffer them, which is why they are considered a severe public health problem.
- Currently, there is ample scientific evidence regarding the usefulness of care models and interventions that prevent and treat osteoporosis.
- An international, multidisciplinary expert group and representatives of multiple scientific societies and patient associations (annex I) have published a decalogue of recommendations to follow to improve care.
- This document intends to disseminate the recommendations, with the aim to facilitate their implementation.
- Healthcare professionals, patients, healthcare managers, politicians and society in general have the obligation of knowing, developing and implementing these recommendations to improve quality of care.

## Supplementary Information

Below is the link to the electronic supplementary material.

ESM 1 (DOCX 18.1 KB)
