# Supplementary material for: Recommendations for the prevention of fragility fractures: a consensus from international experts and Ibero-American scientific societies
Source: Arch Osteoporos. 2025 Jun 12;20(1):76. doi: 10.1007/s11657-025-01551-2 (PMC12162770; doi:10.1007/s11657-025-01551-2)
Supplement: Supplementary file 1 — (DOCX 18.1 KB) [file 11657_2025_1551_MOESM1_ESM.docx]

**Recommendations for the prevention of fragility fractures: a consensus from international experts and Ibero-American scientific societies.**

Supplement 1: Signatories of the Ibero-American multidisciplinary consensus on the prevention of fragility fractures.

| **Association (Acronym) / Translation** | **Country / Region** | **Signatory** |
| --- | --- | --- |
| *Asociación Argentina para el Estudio del Climaterio* (AAPEC) / Argentinian Association on the Study of Menopause | Argentina | Pablo Carpintero Benítez (President) |
| *Academia de Ciencias Médicas de Bilbao* (ACMB) / Academy of Medical Sciences of Bilbao, Spain | Spain | José Luis Neyro (Section President) |
| *Asociación Española con la Osteoporosis y la Artrosis* (AECOSAR) / Spanish Association (of patients) with Osteoporosis and Osteoarthritis | Spain | José Luis Baquero Úbeda (General Secretary) |
| *Asociación Latinoamericana de Endocrinología* (ALEG) / Latin American Association of Endocrinology | Latin America | Germán Salazar Santos (President of the Scientific Committee) |
| *Academia Mexicana de Geriatría A.C* (AMG) / Mexican Academy of Geriatrics | Mexico | Vianey Garzón López (Delegate) |
| *Asociación Colombiana de Menopausia* (ASOMENOPAUSIA) / Colombian Menopause Association | Colombia | Janire Elisa Buelvas (President) |
| *Fundación Navarro Viola* (FNV) / *Navarro Viola Foindation* | Argentina | Magdalena Saieg (Executive Director) |
| *Fundación Trauma* (FT) / Trauma Foundation | Argentina | Laura Bosque (Executive Director) |
| Fragility Fracture Network Denmark (FFN-Denmark) | Denmark | Henric Palm (President) |
| Fragility Fracture Network Greece (FFN-Greece) | Greece | Christos Lionis (President) |
| Fragility Fracture Network Portugal (FFN-Portugal) | Portugal | Bruno Carvalho (Vice President) |
| *Fundación Hispana de Osteoporosis y Enfermedades del Metabolismo Óseo* (FHOEMO) / Hispanic Foundation of Osteoporosis and Bone Mineral Metabolism Disease | Spain | Santiago Palacios Gil Antuñano (President) |
| Osteoarthritis Foundation International (OAFI) | Spain | José Luis Baquero Úbeda (Coordinator of Corporate Social Responsibility, Institutional Relations and Access) |
| *Red Argentina de Fracturas de Cadera* (RAFCA) / Argentinian Network of Hip Fractures | Argentina | María Diehl (National Coordinator) |
| *Registro Nacional de Fracturas de Cadera* (RNFC) / Spanish National Hip Fracture Registry | Spain | Pilar Sáez López (National Coordinator)  Cristina Ojeda Thies (International Liaison) |
| *Sociedad Española de Calidad Asistencial* (SECA) / Spanish Society Of Quality of Care | Spain | Manuel Santiñá Vila (Past President) |
| *Sociedad Española de Cirugía Ortopédica y Traumatología* (SECOT) / Spanish Society of Orthopedic Surgery and Traumatology | Spain | Francisco Baixauli García (Vice President) |
| *Sociedad Española de Anestesiología, Reanimación y Terapéutica del Dolor* (SEDAR) / Spanish Society fo Anesthesiology, Reanimation and Pain Therapy | España | César Aldecoa Santullano (Vice President) |
| *Sociedad Española de Directivos de la Salud* (SEDISA) / Spanish Society of Health Directors | Spain | José Soto Bonel (President) |
| *Sociedad Española de Endocrinología y Nutrición* (SEEN) / Spanish Society of Endocrinology and Nutrition | Spain | María Cortés Berdonces (Coordinator of the Group “Bone and Mineral Metabolism”) |
| *Sociedad Española de Fracturas Osteoporóticas* (SEFRAOS) / Spanish Society of Fragility Fractures | Spain | Concepción Cassinello Ogea (President)  Leonor Cuadra (Vice President)  Teresa Pareja (Past President) |
| *Sociedad Española de Geriatría y Gerontología* (SEGG) / Spanish Society of Geriatrics and Gerontology | Spain | José Augusto García Navarro (President)  Patricia Ysabel Condorhuamán Alvarado (Coordinator, Osteoporosis Group)  Francisco José Tarazona-Santabalbina (Delegate, Clinical Division) |
| *Sociedad Española de Investigación Ósea y del Metabolismo Mineral* (SEIOMM) / Spanish Society of Bone and Mineral Metabolism Research | Spain | Guillermo Martínez Díaz-Guerra (President)  Mercedes Giner García (Vice President) |
| *Sociedad Española de Medicina Geriátrica* (SEMEG) / Spanish Society of Geriatric Medicine | Spain | Nuria Fernández Martínez (Vice President) |
| *Sociedad Española de Médicos de Atención Primaria* (SEMERGEN) / Spanish Society of Primary Care | Spain | Rafael Manuel Micó Pérez (First Vice President) |
| *Sociedad Española de Médicos Generales y de Familia* (SEMG) / Spanish Society of General and Family Physicians | Spain | José Carlos Bastida Calvo (Coordinator, Group “Osteoarticular Disease / Osteoporosis”) |
| *Sociedad Española de Medicina Interna* (SEMI) / Spanish Society of Internal Medicine | Spain | Rosa Arboiro Pinel (Coordinator, Group “Osteoporosis and Mineral Metabolism”) |
| *Sociedad Española de Reumatología* (SER) / Spanish Society of Rheumatology | Spain | Antonio Naranjo Hernández (Delegate) |
| *Sociedad Española de Rehabilitación y Medicina Física* (SERMEF) / Spanish Society of Rehabilitation and Physical Medicine | Spain | Blanca Mur Molina (Delegate)  Carolina de Miguel Benadiba (President) |
| *Sociedad Iberoamericana de Osteología y Metabolismo Mineral* (SIBOMM) / Iberian-american Society of Osteology and Mineral Metabolism | Ibero- America | José Luis Neyro (President) |
| Asociación Vasca de Geriatría y Gerontología (ZAHARTZAROA) / Basque Association of Geriatrics and Gerontology | Spain | Naiara Fernández Gutiérrez (Vice President, Clinical Area) |
